# Single-nucleotide polymorphism-gene intermixed networking reveals co-linkers connected to multiple gene expression phenotypes

**DOI:** 10.1186/1753-6561-1-s1-s45

**Published:** 2007-12-18

**Authors:** Bin-Sheng Gong, Qing-Pu Zhang, Guang-Mei Zhang, Shao-Jun Zhang, Wei Zhang, Hong-Chao Lv, Fan Zhang, Sa-Li Lv, Chuan-Xing Li, Shao-Qi Rao, Xia Li

**Affiliations:** 1 Department of Bioinformatics, Harbin Medical University, Harbin 150081, People's Republic of China; 2 Department of Computer Science, Harbin Institute of Technology, Harbin 150080, People's Republic of China; 3 The First Clinical College, Harbin Medical University, Harbin 150081, People's Republic of China; 4 Departments of Molecular Cardiology and Cardiovascular Medicine, Cleveland Clinic Foundation, 9500 Euclid Avenue, Cleveland, Ohio 44195, USA; 5 Department of Bioinformatics, Capital University of Medical Sciences, Beijing 100084, People's Republic of China

## Abstract

Gene expression profiles and single-nucleotide polymorphism (SNP) profiles are modern data for genetic analysis. It is possible to use the two types of information to analyze the relationships among genes by some genetical genomics approaches. In this study, gene expression profiles were used as expression traits. And relationships among the genes, which were co-linked to a common SNP(s), were identified by integrating the two types of information. Further research on the co-expressions among the co-linked genes was carried out after the gene-SNP relationships were established using the Haseman-Elston sib-pair regression. The results showed that the co-expressions among the co-linked genes were significantly higher if the number of connections between the genes and a SNP(s) was more than six. Then, the genes were interconnected via one or more SNP co-linkers to construct a gene-SNP intermixed network. The genes sharing more SNPs tended to have a stronger correlation. Finally, a gene-gene network was constructed with their intensities of relationships (the number of SNP co-linkers shared) as the weights for the edges.

## Background

It is increasingly recognized through genomic studies that genes regulated via the same mechanisms are likely to have similar mRNA expression profiles, for example, by sharing the same transcriptional factors, or pathways, or any unknown but important factor. Several investigators have provided indirect evidence for this hypothesis by clustering genes according to their mRNA expression profiles [1]. Thus, it may be a feasible strategy to search for co-regulated gene clusters from the correlated genes linked to a common SNP(s) as the shared factor. The relationship between the co-expression and the shared factor(s) (which we call co-linkers) has not been directly tested or quantified on a large scale previously because it is difficult to provide a reliable estimate to measure such relationships using a small number of genes and factors. In this study, we were very interested in identifying such genetic factors (for instance SNPs). A SNP co-linker *cis*- or *trans*-linked with a number of genes may indicate that it is either a functional polymorphism or close to an underlying genetic co-factor nearby that is able to modulate these genes. Today, both genome-wide gene expression and SNPs can be measured at the same time, which allows identification of such *cis*- and *trans*-acting loci, often called (pleiotropic) expression quantitative trait loci (eQTLs) at the "omics" scale. By treating gene expressions as quantitative traits and SNPs as genomic landmarks, the analysis can proceed in the same (or extended) manner as mapping genetic loci for physiologic or clinical traits [2].

In this study, following the conventional linkage analysis for identification of the "susceptibility" SNP/loci for each gene, we further identified these SNP co-linkers by measuring the strength of the relationship between the linked genes and the co-linker. After finding SNP co-linkers, the SNP-gene intermixed network could be further expanded by linking two genes if they shared a common SNP co-linker. We used Haseman-Elston sib-pair linkage analysis to establish gene-SNP linkages. Then, hub genes and SNPs were identified by their high degree of connectivity. Finally, a SNP-gene intermixed network was constructed.

## Methods

### Data preparation

In this study, we used data for Problem 1 from Genetic Analysis Workshop 15 (GAW15), which provided expression levels of 3554 genes in lymphoblastoid cells from fourteen three-generation Centre d'Etude du Polymorphisme Humain (CEPH) Utah families. Genotypes of 2882 autosomal and X-linked SNPs were also included [3]. For the sib-pair linkage analysis, allele frequencies of each SNP locus were estimated by using a maximum-likelihood method incorporated in the FREQ program of S.A.G.E. (Version 5.3) [4], and the identical-by-decent (IBD) data were produced by the GENIBD program in S.A.G.E. [4]. Due to the problem for multipoint IBD computations of very dense SNPs, we performed only single-point IBD computations.

### Sib-pair linkage analysis

We used the SIBPAL program in S.A.G.E. for sib-pair linkage analysis [4]. This is a model-free linkage analysis program based on the Haseman-Elston regression test that models trait data from full-sib pairs as functions of marker allele sharing IBD. Denote the *j*^th ^sib-pair with the subscript *ii*', and define the mean transcriptional expression for a gene as:

$$\bar{x}=\frac{1}{2N}\sum_{j=ii^{\prime }=1}^{N}\left(x_{i}+x_{i^{\prime }}\right),$$

where the summands were the gene expression values measured on *N *sib pairs. Then the dependent variable for the *j*^th ^pair was

*y*_*j *_= (*x*_*i *_- $$\bar{x}$$)(*x*_*i' *_- $$\bar{x}$$).

The basic regression model we fitted was of the form:

*y *= *α *+ *β*_*h*_$$\hat{\pi }$$_*h *_+ *ε*,

where *α *was the intercept, and *β*_*h *_and $\hat{\pi }$_*h *_were the total genetic variance due to the *h*^th ^SNP marker and the estimated IBD at the marker respectively, and *ε *was the residual error. This regression model was used to establish the gene-SNP linkages.

### Constructing a gene-SNP intermixed network

**Figure 1:**
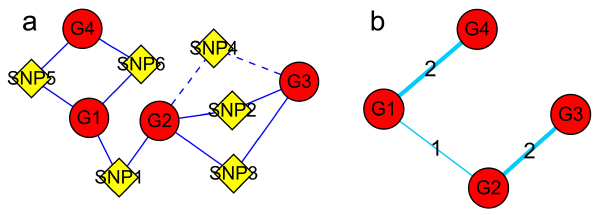
**A graphical illustration for constructing agene-SNP intermixed network and a gene-gene network**. In the graph, a red node represents a gene and a yellow node a SNP. a) A gene-SNP intermixed network. A SNP-gene pair is connected by either a solid or a dashed line. The dashed line denotes the scenario in which a SNP co-linker is in strong LD with other SNP(s) (e.g., SNP2 and SNP4); b) A gene-gene network extracted from the gene-SNP intermixed network. The number on a line shows the intensity of relationship between the two genes.

A gene-SNP network was constructed using the identified relationships between genes and SNPs, where genes and SNPs were two types of nodes. Two nodes were connected using an edge if a relationship between them was presented (Fig. 1a). Some nodes that had markedly more connections with other nodes were defined as "hub nodes", including "hub genes" and "hub SNPs". Hub genes or hub SNPs, which may have more important functions or key roles in some biological processes or pathways, were subjected to further investigations.

### Exploring the expression pattern between genes linked to a common SNP locus

The genes that were linked to a common SNP(s) might have correlated mRNA expression profiles because they share a genetic factor. Let *G*_*i *_be a group of *n *genes that were linked to a common SNP (identified via the previous sib-pair linkage analysis). The hypothesis tested (H_t_) was whether the genes in *G*_*i *_were significantly co-expressed by comparing it with the null model (H_0_) that depicts the random expression patterns among groups of genes of the same size as *G*_*i*_. The averaged Pearson's correlation coefficient ($r_{G_{i}}$) was used as the metric to measure the transcription consensus:

$$r_{G_{i}}=\frac{\sum_{i,j=1,i<j}^{n}2r_{ij}}{n(n-1)},$$

where *r*_*ij *_was the Pearson's correlation coefficient between *i*^th ^and *j*^th ^genes. A gene could be clustered into different functional groups if it was linked to multiple SNP co-linkers. A permutation approach was used to determine the consensus threshold for *G*_*i*_. We randomly sampled *n *genes from the original whole gene pool and calculated the averaged correlation coefficient between the *n *genes. The same process was repeated 100,000 times and the 95% quantile was then defined as the empirical threshold that corresponds to the type I error of 0.05 [5].

### Extracting a gene-gene network

Gene expressions are often influenced by multiple factors (e.g., transcription factors, reporters, enhancers, modifiers, and so on). The more factors two genes share, the closer the relationship they tend to have. However, the strong linkage disequilibrium (LD) among the SNP co-linkers might influence the result. To investigate the issue, we assessed the LD for all SNP pairs by implementing a likelihood ratio test using the software Arlequin [6], whose empirical distribution was obtained by a permutation procedure [7]. We found that LD had a small influence on the result (only 4.8% of gene pairs were co-linked with a SNP pair or block of a significant LD (*p *< 0.05)). As shown in Figure 1b, we defined two genes to be connected if they shared at least one linked SNP and the number of co-linked non-significant LD SNPs for the two genes was used to describe the intensity of their relationship (considering a SNP pair of a significant LD as a single SNP). A gene-gene network was then constructed according to the newly identified relationships with their intensities of relationship as the weights for the edges.

## Results

### Gene-SNP relationships

We found a total of 4727 gene-SNP linkage pairs at the significance level of 10^-7 ^after multiple test correction using Bonferroni's method. There were 60 pairs defined as *cis*-acting where the SNPs localized within the extended 10-Mb region of the paired genes, and all the remaining were *trans*-acting.

### Gene-SNP intermixed network and hubs

**Figure 2:**
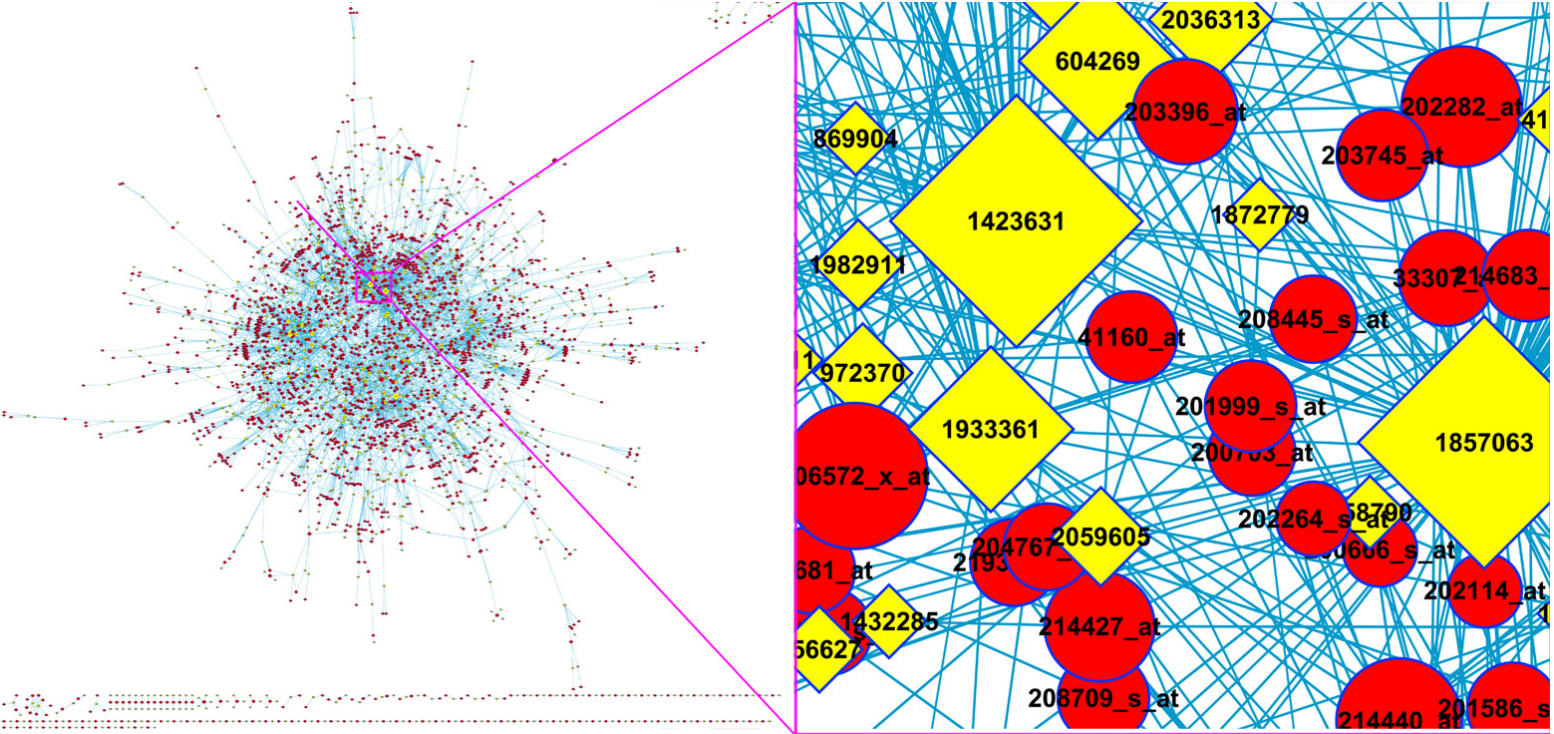
**Gene-SNP intermixed network**. The size of each node is scaled by its degree of connectivity. Hub nodes thus are readily viewed due to their larger size.

A gene-SNP network was constructed from the above gene-SNP linkages. Genes and SNPs had 2.4 connections on average. Some genes and SNPs had a significantly larger number of relationships than the average. These genes and SNPs were defined as hub nodes (either hub genes or hub SNPs) as illustrated in Figure 2. Inferring from their topological roles that these genes would likely be lethal if mutated, we expect they might have more important function(s) in the underlying pathways, but this would have to be verified in wet-lab experiments for their pleiotropic influences on multiple genes. The top 1% hub genes or hub SNPs are listed in Tables 1 and 2, respectively.

**Table 1:** The top 1% genes of a high degree of connectivity

Affy ID	Degree
201209_at	21
219424_at	19
212186_at	18
206572_x_at	18
200743_s_at	17
215171_s_at	17
206492_at	16
212724_at	15
209790_s_at	15
203711_s_at	14
205292_s_at	13
204558_at	13
209112_at	12
200853_at	12
208901_s_at	12
209785_s_at	12
203439_s_at	11
210981_s_at	11
202209_at	11
217985_s_at	11
201714_at	11
214440_at	10
218036_x_at	10
210950_s_at	10
210640_s_at	10
201111_at	10
202546_at	10
204982_at	10
204348_s_at	10

**Table 2:** The top 1% SNPs of a high degree of connectivity

SNP ID	Degree	Co-expression measure	*p*-Value
1857063	114	0.3650	<0.0001
700543	62	0.3002	<0.0001
1430952	52	0.2106	0.0031
1423631	51	0.3069	<0.0001
1468059	48	0.3821	<0.0001
1079635	44	0.2386	<0.0001
1478292	36	0.2451	0.0001
1945465	35	0.2124	0.0104
897653	33	0.2144	<0.0001
889512	30	0.2618	<0.0001
2046725	30	0.2808	<0.0001
728284	28	0.2277	0.0040
2052547	28	0.3641	<0.0001
1077647	28	0.3375	<0.0001

### Co-expression pattern between genes linked to a common SNP locus

**Figure 3:**
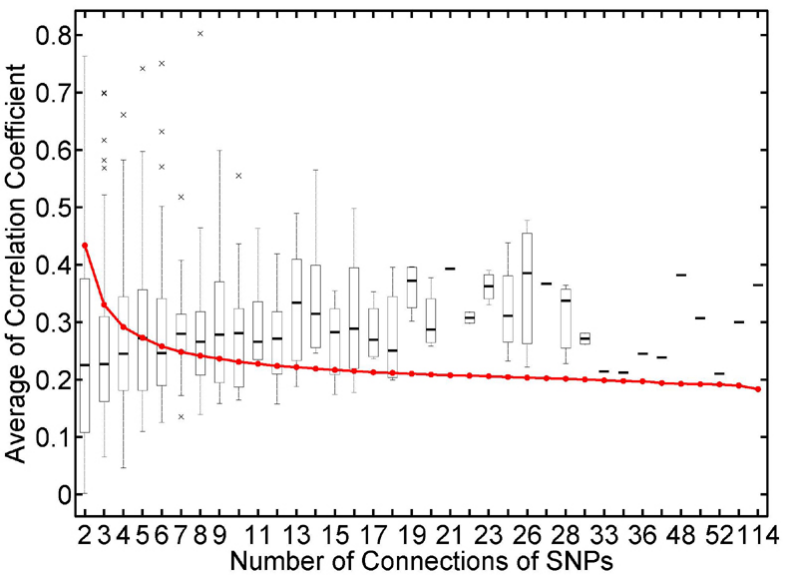
**Intensity of co-expression of genes linked to acommon SNP(s)**. The red line, generated by 100,000 permutations for each sized (i.e., the number of connections of the SNP co-linker) gene cluster, shows the empirical threshold for the average Pearson's correlation coefficient among the genes in the cluster, corresponding to the type I error of 0.05.

We used the averaged correlation coefficient between co-linked genes as their co-expression measure. Table 2 lists the results for the top 1% SNP co-linkers, and the *p*-values correspond to the empirical values derived from the null distribution generated by 100,000 permutations. In this study, we found that the genes among a group having number of connections >18 were significantly co-expressed. If the number of connections dropped down between 18 and 7, co-expressions of most gene clusters were less significant. But, if the number dropped down below 7, co-expression might be occurring by chance (Fig. 3).

### Gene-gene network

**Figure 4:**
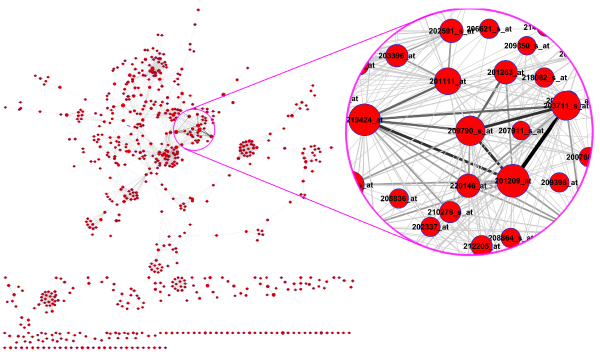
A reconstructed gene-gene interaction network.

The gene-gene network (Fig. 4) was extracted from the gene-SNP intermixed network (Fig. 2). The network can obviously be divided into several agglomerate subnets (illustrated in Fig. 4). We used the number of co-linked SNPs of each gene pairs as their interacting power (Pi). Gene pairs with a high Pi may have a similar function(s) because potentially they share more common factors. However, this was not investigated further.

## Discussion

In our study, first we used a univariate method to identify gene-SNP relationships for each gene expression phenotype separately. After finding the pool of all gene-SNP relationships, we constructed the SNP-gene intermixed network and further gene-gene network based on the concept of sharing a factor(s). Alternatively, one may consider using purely multivariate modelling to include all the genes, but this is not practical yet due to the prohibitive computing demand. One may also consider several data reduction techniques such as principal-component analysis as seen in several applications in this workshop. From both theoretical and applied views, all the methods have their own merits and weaknesses in analyzing modern high-dimension data. Further comparisons between different approaches are expected to provide novel methodological insights.

Although we identified gene-SNP relationships using very strict criteria, it is likely that the resulting gene-SNP intermixed network (or the further derived gene-gene network) includes some noise links. Hub SNPs or hub genes, which are expected to play key roles in maintaining the underlying biological networks in a working condition, should be further studied either computationally or using molecular biology techniques prior to applying these hub SNPs or hub genes in more practical settings.

## Conclusion

The proposed bioinformatics approach, which attempts to identify co-regulated gene clusters from the correlated genes linked to a common SNP(s) as the shared factor, has been demonstrated via analysis of the GAW15 Problem 1 data, to be a promising and feasible strategy to identify transcriptional QTLs (hub SNPs) and to elucidate the underlying transcriptional genetic networks on the global scale. However, further studies, including topological and functional evaluation and validation of the resulted networks using more data sets and large-scale simulations are warranted.

## Competing interests

The author(s) declare that they have no competing interests.
